# Recent Acquisition of *Helicobacter pylori* by Baka Pygmies

**DOI:** 10.1371/journal.pgen.1003775

**Published:** 2013-09-19

**Authors:** Sandra Nell, Daniel Eibach, Valeria Montano, Ayas Maady, Armand Nkwescheu, Jose Siri, Wael F. Elamin, Daniel Falush, Bodo Linz, Mark Achtman, Yoshan Moodley, Sebastian Suerbaum

**Affiliations:** 1 Institute of Medical Microbiology and Hospital Epidemiology, Hannover Medical School, Hannover, Germany; 2 Konrad Lorenz Institute for Ethology, Department of Integrative Biology and Evolution, University of Veterinary Medicine Vienna, Vienna, Austria; 3 Department of Endoscopy, Republic Hospital No. 1, Kyzyl City, Republic of Tuva, Russia; 4 Ministry of Public Health, Division of Operational Research, Yaoundé, Cameroon; 5 International Institute for Applied Systems Analysis, Laxenburg, Austria; 6 El Razi College of Medical and Technology Sciences, Khartoum, Sudan; 7 Department of Statistics, University of Oxford, Oxford, England; 8 Max Planck Institute for Infection Biology, Department of Molecular Biology, Berlin, Germany; 9 Department of Biochemistry and Molecular Biology, Pennsylvania State University, University Park, Pennsylvania, United States of America; 10 Environmental Research Institute and Department of Microbiology, University College Cork, Cork, Ireland; 11 Warwick Medical School, University of Warwick, Coventry, United Kingdom; University of Oxford, United Kingdom

## Abstract

Both anatomically modern humans and the gastric pathogen *Helicobacter pylori* originated in Africa, and both species have been associated for at least 100,000 years. Seven geographically distinct *H. pylori* populations exist, three of which are indigenous to Africa: hpAfrica1, hpAfrica2, and hpNEAfrica. The oldest and most divergent population, hpAfrica2, evolved within San hunter-gatherers, who represent one of the deepest branches of the human population tree. Anticipating the presence of ancient *H. pylori* lineages within all hunter-gatherer populations, we investigated the prevalence and population structure of *H. pylori* within Baka Pygmies in Cameroon. Gastric biopsies were obtained by esophagogastroduodenoscopy from 77 Baka from two geographically separated populations, and from 101 non-Baka individuals from neighboring agriculturalist populations, and subsequently cultured for *H. pylori*. Unexpectedly, Baka Pygmies showed a significantly lower *H. pylori* infection rate (20.8%) than non-Baka (80.2%). We generated multilocus haplotypes for each *H. pylori* isolate by DNA sequencing, but were not able to identify Baka-specific lineages, and most isolates in our sample were assigned to hpNEAfrica or hpAfrica1. The population hpNEAfrica, a marker for the expansion of the Nilo-Saharan language family, was divided into East African and Central West African subpopulations. Similarly, a new hpAfrica1 subpopulation, identified mainly among Cameroonians, supports eastern and western expansions of Bantu languages. An age-structured transmission model shows that the low *H. pylori* prevalence among Baka Pygmies is achievable within the timeframe of a few hundred years and suggests that demographic factors such as small population size and unusually low life expectancy can lead to the eradication of *H. pylori* from individual human populations. The Baka were thus either *H. pylori*-free or lost their ancient lineages during past demographic fluctuations. Using coalescent simulations and phylogenetic inference, we show that Baka almost certainly acquired their extant *H. pylori* through secondary contact with their agriculturalist neighbors.

## Author Summary

Genetic analyses of *Helicobacter pylori* have illuminated human migrations and the history of human infection by these bacteria. Both humans and *H. pylori* originated in Africa, and have been intimately associated for at least 100,000 years. We hypothesized that communities who still live in relative isolation might provide further details about the evolutionary history of *H. pylori* in Africa. We therefore investigated *H. pylori* within Baka Pygmies of southeast Cameroon, who live as hunter-gatherers in the tropical rainforest, and compared those bacteria to *H. pylori* from neighboring farming populations of non-Baka ethnicities. Unexpectedly, Baka Pygmies were much less commonly infected (20.8%) than the non-Baka (80.2%). *H. pylori* from hunter-gatherers and agriculturalists were genetically very similar and ancient *H. pylori* lineages were not identified in Baka. We used an epidemiological model to show that demographic factors including small population size and low life expectancy can account for the low infection rate among Baka Pygmies, and that this low rate could have been attained within a few hundred years of secondary contact with their neighbors. We also suggest that the ancestors of the Baka Pygmies were initially *H. pylori*-free or that their ancestral bacteria have been lost through past demographic fluctuations.

## Introduction

*Helicobacter pylori* is a major gastric pathogen that infects more than half the world's human population. Infection is almost always life-long, generally acquired during early childhood and results in gastric inflammation, which remains asymptomatic in most individuals. 10–15% of infected individuals develop gastric or duodenal ulcers, and infection with *H. pylori* is also a cause of gastric cancer and mucosa-associated lymphoid tissue (MALT) lymphoma [1]. Transmission within families plays a dominant role in areas with better sanitary conditions, but extra-familial horizontal transmission is typical in rural areas of the developing world [2], [3]. As a result, developing countries have infection rates that are much higher than the global average, reaching >90% in many areas [4]–[7].

**Figure 1:**
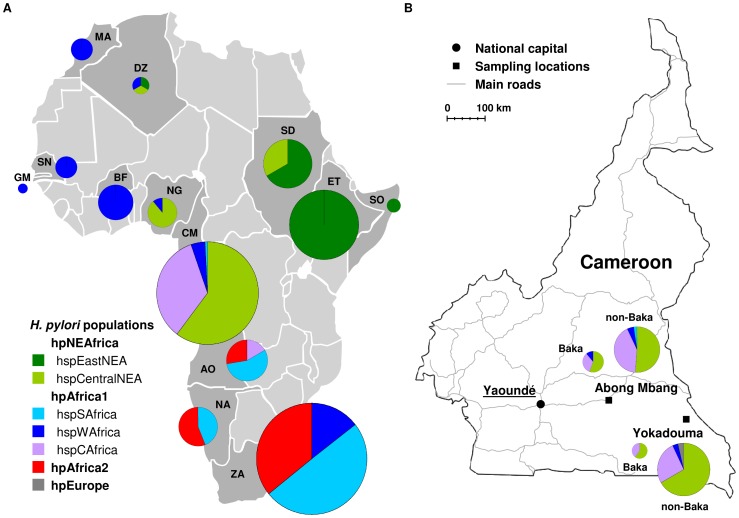
The sources of African *H. pylori* isolates. (A) The proportions of haplotypes from the different African *H. pylori* populations at each sampling location are displayed as pie charts, whose size is proportional to the total number of haplotypes. Colors indicate STRUCTURE population assignments. All hpAfrica1 and hpNEAfrica isolates indicated were used in the corresponding STRUCTURE analyses. Country code: DZ, Algeria; AO, Angola; BF, Burkina Faso; CM, Cameroon; ET, Ethiopia; GM, Gambia; MA, Morocco; NA, Namibia; NG, Nigeria; SN, Senegal; SO, Somalia; ZA, South Africa; SD, Sudan. (B) The distributions of the Cameroonian haplotypes at the two sampling locations are displayed as pie charts. The size corresponds to the total number of haplotypes. The Baka villages are located at distances of 18–19 km from Abong Mbang and 6–14 km around Yokadouma, respectively. The non-Baka participants lived either in Abong Mbang or Yokadouma or in villages located within a distance of less than 50 km from Abong Mbang, or 36 km from Yokadouma.

The association of *H. pylori* with humans is ancient, dating back at least 100,000 years (100 kyr) [4]. Africa is the ancestral home of humans and *H. pylori* alike, and three distinct African populations have been identified within this bacterial species [8]. Infected anatomically modern humans carried *H. pylori* with them when they left Africa about 60,000 years ago (60 kya) [8], and diverse demographic and selective pressures subsequently resulted in four additional, geographically-associated populations in Eurasia, Sahul and the Americas [8]–[10]. The genetic diversity of *H. pylori* reflects historical human demographic events, including the peopling of the Pacific [10], the colonization of the Americas [9] and migrations in Iran [11], northern India [12], Malaysia [13] and Southeast Asia [14], [15]. Until recently, however, little attention was paid to demographic movements during early human history in Africa. The three *H. pylori* populations that are indigenous to Africa have been designated hpNEAfrica, hpAfrica1, and hpAfrica2 [8], [9] (Figure 1A). hpAfrica1 has been subdivided into two subpopulations: hspSAfrica, found mainly in South Africa, and hspWAfrica, which is found in both West and South Africa. hpNEAfrica was isolated from Nilo-Saharan speakers in Ethiopia, Somalia, Sudan, Nigeria and Algeria. hpAfrica2, the most genetically distinct of all *H. pylori* populations, was isolated only from individuals in South Africa, Namibia and southern Angola [4], [9], [16].

The existence of these multiple populations of *H. pylori* reflects diversification in Africa after its initial acquisition by humans at least 100 kya. Thereafter, *H. pylori* diverged into two super-lineages, one which became hpAfrica2, and a second which eventually diversified into all other extant populations. hpAfrica2 evolved within the click-speaking San [4], a group of hunter-gatherers indigenous to southern Africa who represent one of the deepest branches of the human population tree [17]–[22]. Genome-wide analyses of numerous human populations have revealed that other hunter-gatherer populations such as Pygmies, Sandawe and Hadza also branched early in African prehistory, suggesting a common and ancient origin for hunter-gatherers [20], [23], [24]. Pygmy hunter-gatherers diverged from the ancestors of contemporary agriculturalist Africans about 56–70 kya [25], [26]. Population splits within each hunter-gatherer group, however, are more recent. Molecular data suggest that the San split into two groups only 32–47 kya [4], [27] whereas Pygmies separated into ancestral Western and Eastern populations ∼20 kya [25] (see Figure S1).

From a bacterial perspective, only the hpAfrica2 super-lineage has been found to be associated with hunter-gatherers thus far. The populations hpNEAfrica and hpAfrica1 comprise the African component of the second *H. pylori* super-lineage, sharing a common ancestor 36–52 kya [4]. Therefore they may reflect more recent human demographic events in Africa. We find hpNEAfrica to be endemic among eastern Nilo-Saharan speakers in northeast Africa [8]. This language group is thought to have originated in northeast Africa, expanding westward during the humid Holocene period (6–9 kya) when the central Sahara and beyond was inundated with rivers and wetlands [28] (Figure S1). Since only few hpNEAfrica strains have been isolated from elsewhere in Africa, it has remained unclear whether hpNEAfrica strains from Nigeria and Algeria represent an ancient westward expansion of Nilo-Saharans or modern migrations. hpAfrica1 appears to be associated with the more recent sub-Saharan migrations of a highly successful group of agriculturalists whose languages belong to a closely related language family commonly referred to as Bantu - officially the Benue-Congo subfamily of the Niger-Congo language family. Population expansions of Bantu speakers from their homeland in modern Cameroon/Nigeria spread their languages and agriculture through most of sub-Saharan Africa in the last 3–6 kyr [29]. Bantu speakers are thought to have migrated in two waves that skirted the dense tropical rainforests, down the west coast towards southern Africa and to the eastern side of Africa followed by further southward migrations [30], [31] (Figure S1), although the details remain controversial [31], [32]. The west and south African subpopulations within hpAfrica1 might parallel these human migrations [9].

Considering the ancient origins of Pygmy mitochondrial and Y-chromosome lineages, and by analogy with the association between hpAfrica2 and San hunter-gatherers, we hypothesized that Pygmies might be infected with a currently unknown, Pygmy-specific population of *H. pylori* derived from one of the two super-lineages. Similar to the San, we also anticipated that Pygmies may have acquired populations of *H. pylori* from their agriculturalist neighbors, with whom they have been in contact for 3–6 kyr [33], [34], and from whom they acquired the Bantu language. We therefore tested these hypotheses in a population genetic study of the *H. pylori* infecting the Baka, a subgroup of the Western Pygmies from two populations in Cameroon. Baka inhabit the rainforests of Cameroon, Congo and Gabon and constitute one of the largest of all Pygmy groups, with a population size of 30–40,000 [35]. The prevalence and population structure of the *H. pylori* infecting the Baka and other Pygmies was previously unknown. Their Bantu-speaking, agriculturalist neighbors all have high *H. pylori* infection rates [36], but again the population structure of those bacteria was unknown. We therefore also screened *H. pylori* from neighboring non-Baka of multiple ethnic groups in order to quantify the levels of bacterial gene flow between hunter-gatherers and agriculturalists.

We obtained several interesting results: 1) the prevalence of *H. pylori* infection among Baka Pygmies is much lower than among neighboring non-Baka agriculturalists, 2) *H. pylori* from the Baka did not define a new population, 3) Baka acquired their *H. pylori* infections from non-Baka neighbors, 4) acquisition of *H. pylori* by the Baka was recent, and post-dated the 3–6 kyr of secondary contact between Baka and non-Baka, 5) the frequency of *H. pylori* infection of the Baka is probably limited by population size and other demographic factors.

## Results

### Infection rates

We analyzed *H. pylori* prevalence in 178 Cameroonians, including 77 Baka Pygmies and 101 non-Baka agriculturalists, by bacterial culture from gastric mucosal biopsies obtained through esophagogastroduodenoscopy (Table 1). Among the Baka, 46.8% were male; the mean age of the Baka participants was 38.9 years. Among non-Baka, 21.8% were male, with a mean age of 46.0 years. Similar inter-sample age differences were present for males and females. We found a high prevalence of *H. pylori* infection (81/101; 80.2%) within the non-Baka (Table 1), which is concordant with infection rates in Cameroon among patients [5], [36] and asymptomatically infected individuals [37]. However, the infection rate among Baka Pygmies was significantly lower (16/77; 20.8%; Chi-square test: *p*<0.001) than observed among non-Baka, and this low rate was consistent between the two sampling locations, Abong Mbang (10/42; 23.8%) and Yokadouma (6/35; 17.1%), although they are separated by a distance of over 200 km (Figure 1B). Baka women were more frequently infected than men (24.4% *vs.* 16.7%), and non-Baka men were more frequently infected than non-Baka women (90.9% *vs.* 77.2%). However, these differences were not significant (Fisher exact test: *p*>0.05). A multiple logistic regression (controlling for sex and age) suggests that in this sample, non-Baka individuals were about 16 times more likely to be infected than Baka (OR = 16.1, 95% CI 7.3–35.6, *p*<0.001).

**Table 1:** Assignment of Cameroonian *H. pylori* isolates to populations and subpopulations.

Location	Ethnicity	Language subgroup	No. of individuals			No. of unique haplotypes assigned to *H. pylori* population/subpopulation^1^			
			total	*H. pylori*		hpNEAfrica	hpAfrica1		hpEurope
				neg.	pos.		hspWAfrica	hspSAfrica	
***Hunter-Gatherers***			**77**	**61**	**16**	**8**	**6**	**0**	**0**
Abong Mbang	Baka	Ubangi	42	32	10	5	4	0	0
Yokadouma	Baka	Ubangi	35	29	6	3	2	0	0
***Agriculturalists***			**101**	**20**	**81**	**65**	**39**	**1**	**2**
Abong Mbang			41	8	33	22	21	1	0
	Badjoue	Benue-Congo	5	0	5	5	1	0	0
	Bamileke	Benue-Congo	7	1	6	2	10	0	0
	Bassa	Benue-Congo	1	0	1	0	1	0	0
	Betsi	Benue-Congo	1	0	1	1	0	0	0
	Bikele	Benue-Congo	1	0	1	1	0	0	0
	Boulou	Benue-Congo	1	1	0	0	0	0	0
	Kako	Benue-Congo	1	1	0	0	0	0	0
	Maka	Benue-Congo	17	2	15	10	6	1	0
	Menemo	Benue-Congo	1	1	0	0	0	0	0
	Moghamo	Benue-Congo	1	1	0	0	0	0	0
	Pompom	Benue-Congo	1	0	1	0	1	0	0
	Wimboum	Benue-Congo	1	0	1	0	2	0	0
	Yebekolo	Benue-Congo	2	1	1	1	0	0	0
	Zime	Benue-Congo	1	0	1	2	0	0	0
Yokadouma			60	12	48	43	18	0	2
	Bamileke	Benue-Congo	7	1	6	0	8	0	0
	Bapele	Benue-Congo	1	0	1	0	1	0	0
	Baya	Ubangi	2	0	2	4	0	0	0
	Bimou	Benue-Congo	9	1	8	9	1	0	2
	Bogandou	Benue-Congo	3	1	2	1	2	0	0
	Boman	Benue-Congo	2	2	0	0	0	0	0
	Boulou	Benue-Congo	1	0	1	0	1	0	0
	Foulbe	Atlantic	1	0	1	1	0	0	0
	Kako	Benue-Congo	3	0	3	3	0	0	0
	Konabembe	Benue-Congo	7	2	5	5	1	0	0
	Maka	Benue-Congo	1	0	1	2	0	0	0
	Mvonvon	Benue-Congo	21	4	17	17	4	0	0
	Ndjem	Benue-Congo	1	1	0	0	0	0	0
	Toupouri	Adamawa	1	0	1	1	0	0	0
**Total**			**178**	**81**	**97**	**73**	**45**	**1**	**2**

1 Seven of the ten identical haplotypes were isolated from individuals of different ethnicity and count more than once, thus, the total number of isolates in this table is 121 instead of 113.

Endoscopic evaluations revealed that 80% of all study participants showed evidence of gastroduodenal pathology (Table S1). None of the observed pathologies showed a significant correlation with *H. pylori* infection status, in agreement with prior results from developing countries [6]. Moreover, the prevalence of pathologies was not significantly different between Baka and non-Baka populations, despite their different *H. pylori* infection rates. However, the relatively small number of individuals sampled and the non-randomized design of the study preclude solid conclusions about the associations between *H. pylori* and pathology as observed by endoscopy.

In total, 191 *H. pylori* isolates were cultured from all biopsies, and seven MLST housekeeping gene fragments [38] were sequenced from each isolate. An alignment of the concatenated sequences (3,406 bp) revealed 113 distinct haplotypes. Ten haplotypes were shared among more than one individual, the most common of which was found in five individuals, including three Baka Pygmies and two non-Baka individuals. Isolates from *antrum* and *corpus* differed in 31 individuals, although most of these strain pairs varied only slightly. There was only one pair of highly divergent strains, which is a clear indication of a mixed infection with unrelated strains. Finding identical haplotypes in multiple individuals indicates that transmission is frequent, relative to mutation and recombination rates, especially considering the numbers of strain pairs from single individuals that differed slightly in their haplotype sequences.

### Population structure and phylogeography

**Figure 2:**
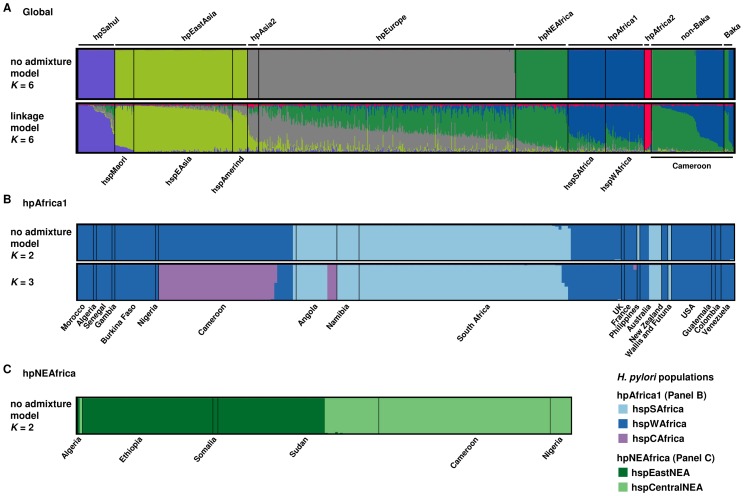
Bayesian population assignments using STRUCTURE 2.0. (A) DISTRUCT plot of the population assignment of Cameroonian *H. pylori* haplotypes in a global reference dataset as determined by the “no admixture model” (*K* = 6) and the “linkage model” (*K* = 6). Each bacterial isolate is depicted by a thin vertical line, which is divided into *K* colored segments representing the membership coefficients in each cluster. Black lines separate isolates of different populations/subpopulations. Populations are labeled above the figure, subpopulations below the figure. Cameroonian non-Baka and Baka *H. pylori* haplotypes are shown on the right side of the plot. (B) DISTRUCT plots of the subpopulation assignment of *H. pylori* hpAfrica1 haplotypes using the “no admixture model” (*K* = 2, *K* = 3). Strains were grouped together according to their geographical source. (C) DISTRUCT plot of the population structure of *H. pylori* hpNEAfrica haplotypes according to the “no admixture model” (*K* = 2). Strains were grouped together according to their geographical source.

Bayesian clustering using both the “no admixture model” and the “linkage model” of STRUCTURE [39] assigned most haplotypes to the populations hpNEAfrica (67; 59.3%) or hpAfrica1 (44; 38.9%) (Figure 2A). Two strains isolated from one non-Baka individual were assigned to hpEurope. hpAfrica2 was not isolated nor did any isolate belong to a novel, Pygmy-specific population. The occurrence of hpAfrica1 strains is not surprising because this population has been isolated repeatedly from Bantu speakers [9]. However, isolating hpNEAfrica in Cameroon was unexpected because it has previously only been found in Afro-Asiatic and Nilo-Saharan speakers [8].

The proportions of Baka and non-Baka in our study that were infected with hpNEAfrica or hpAfrica1 strains were very similar (Table 1), as were the relative levels of nucleotide diversity within hpNEAfrica and hpAfrica1 haplotypes from both communities (Table 2). Intra- and inter-population pair-wise haplotype differences did not reject a null hypothesis of panmixia (p(*Ks*) = 0.12) between Baka and non-Baka populations.

**Table 2:** Genetic diversity of hpAfrica1 and hpNEAfrica haplotypes isolated from Baka Pygmies and non-Baka agriculturalists.

*H. pylori* population	Ethnicity	*N*	*h*	*S*	*k*	*π* (SD)
**hpAfrica1**	Baka	7	6	183	76.29	0.02240 (0.00318)
	non-Baka	40	38	336	81.22	0.02385 (0.00082)
**hpNEAfrica**	Baka	10	8	233	80.80	0.02372 (0.00202)
	non-Baka	70	60	440	76.59	0.02249 (0.00064)

*N*, total number of sequences; *h*, number of haplotypes; *S*, Number of polymorphic (segregating) sites; *k*, average number of nucleotide differences; *π*, nucleotide diversity; SD, standard deviation.

Using a composite-likelihood approach to estimate population demographic parameters under an isolation with migration model (Jaatha 2.0) [40], we estimated the time to population split of *H. pylori* between Baka and non-Baka using explicit demographic models of constant population size and exponential expansion (see Methods for details). The composite likelihood for model 1 (−197.4695; constant population size in Baka) was higher than for scenario 2 (−197.5009; exponential growth in both populations), but estimates for time to population split and effective population size (Table 3) were very similar. Gene flow into the Baka population was higher when Baka bacterial population size was assumed to be constant, although confidence limits for both estimates were widely overlapping. Allowing for gene flow since divergence, we inferred a median date for the split between the Baka and non-Baka *H. pylori* of 2,110 and 2,406 years for constant size and expansion models respectively, with combined confidence limits of 856–3,973 years. These analyses indicate that the modern infections of Baka by *H. pylori* began no earlier than 4,000 years ago, soon after Baka came into contact with non-Baka agriculturalists (∼3–6 kya).

**Table 3:** Population demographic parameters for *H. pylori* from Baka Pygmies and non-Baka agriculturalists derived using an isolation-with-migration model and assuming two demographically explicit scenarios.

	*Theta* (θ)	*Timing* (τ)		*Gene flow* (m)		*Growth* (α)		*Recombination* (rho)
**Model 1: Constant Baka population size, exponential growth of non-Baka population**								
	*θ*_1_ (non-Baka)	*τ*	*T* (years)	Baka→non-Baka	non-Baka→Baka	*α*_1_ non-Baka	*α*_2_ Baka (fixed)	*rho*
Median	13.78	0.0006	2,110	0.2145	0.0781	0.0023	0	0.0033
c.i. L	13.76	0.0003	856	0.0292	0.0047	0.0007	0	0.0003
c.i. U	13.87	0.0012	3,981	0.6325	0.2662	0.0062	0	0.0476
**Model 2: Exponential growth of both Baka and non-Baka populations**								
	*θ*_1_ (non-Baka)	*τ*	*T* (years)	Baka→non-Baka	non-Baka→Baka	*α*_1_ non-Baka	*α*_2_ Baka	*rho*
Median	13.78	0.0007	2,406	0.4373	0.0250	0.0008	0.0012	0.0023
c.i. L	13.71	0.0003	983	0.0502	0.0054	0.0004	0.0003	0.0002
c.i. U	13.89	0.0012	3,973	0.9352	0.0910	0.0016	0.0042	0.0018

Population parameter theta (*θ*) = 4*N_1_μ*, where *N_1_* is the effective population size of the reference population (non-Baka) and *μ* is the mutation rate. *t* is the timing parameter in coalescent units, and *T* the time to split between Baka and non-Baka (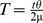). *m* is the fraction of the population replaced by migrants per generation: 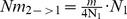 of population 1 (non-Baka) are replaced per generation and 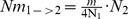 of population 2 (Baka) are replaced per generation. The *θ* estimated by the software refers only to population 1. c.i., confidence interval; L, lower; U, upper.

We also analyzed our Cameroon sample together with other African haplotypes previously assigned to hpAfrica1 or hpNEAfrica, respectively. The most consistent results were obtained for a *K* = 2 scenario within hpAfrica1, wherein 43 haplotypes from Cameroon were assigned to hspWAfrica and only one to hspSAfrica (Figure 2B). Interestingly, at *K* = 3, most Cameroonian haplotypes formed a novel subpopulation, hspCAfrica, together with three haplotypes from Angola. These analyses also provide evidence for genetic structure within hpNEAfrica, for which a *K* = 2 hypothesis was most likely. All haplotypes from Ethiopia (51), Somalia (2) and isolates from Sudan (42/63) and Algeria (1/2) clustered together in an ‘East African’ subpopulation, hspEastNEA, whereas all isolates from Cameroon (67), Nigeria (8) and some isolates from Sudan (21/63) and Algeria (1/2) formed a second ‘Central/West African’ subpopulation which we call hspCentralNEA (Figure 2C).

ClonalFrame was used to further elucidate the substructure of both the hpAfrica1 and hpNEAfrica populations. The resulting genealogies (Figures S2 and S3) highlight the interspersed distribution of Baka *H. pylori* isolates among the non-Baka strains from Cameroon, without Baka-specific groupings. Both phylogenies comprised several clades, all supported in over 80% of the posterior sample, but corresponding only roughly to the subpopulations within hpAfrica1 and hpNEAfrica.

### *H. pylori* infection among Baka Pygmies is recent

We were intrigued by the low frequency with which the Baka were infected by *H. pylori*, which could further corroborate the hypothesis of recent acquisition by the Baka. In order to investigate these observations quantitatively, we implemented an age-structured SI model, building on an existing model of *H. pylori* infection developed by Rupnow *et al.* [41]. In concordance with observed data, the model assumes that infection is life-long once acquired. Unlike in the original model, we did not further subdivide the compartment of infected individuals by severity or pathology. The model population was divided into three age groups: children, youths and adults; and we stipulated that a fraction of individuals are born non-susceptible, in keeping with observations that show that prevalence never attains 100%, even in highly infectious settings [42], and consistent with the implementation of the existing model [41]. Our model includes the following assumptions: 1) The Baka population is both stable and stationary, that is, both the total population size and the fraction in each age class are constant, allowing us to estimate constant birth and age-specific death rates. The assumption of a stable population is consistent with our coalescent simulations above, that show only very slight differences in splitting times between models of constant population size and population expansion (Table 3). 2) Infection does not affect all-cause mortality. This was demonstrated in a recent study [43]. 3) Inter-age class transmission is governed by the characteristics of the less-transmissive partner. This is conservative, but represents an increase in transmission from the Rupnow *et al.* model [41], which assumes that inter-age class transmission is negligible. 4) The population is closed, with no migration or transmission from external sources. This is unlikely in reality, given the close proximity of agriculturalist communities; however, since the Baka are quite reclusive, we consider it a reasonable simplifying assumption. Additionally, since the Baka have only recently (3–6 kya) come into secondary contact with agriculturalists [33], [34], the assumption would likely hold true for Baka populations prior to this time. 5) Survival decreases linearly within age classes. As did the original model, we also assume that transmission is entirely person-to-person. We then parameterized the model according to our best estimates for demographic and transmission parameters among Pygmies (Table 4). Allowing for inter-age class transmission yielded nine transmission constants (β) rather than the three intra-class coefficients used by Rupnow *et al.* [41].

**Table 4:** Base scenario model parameters determined empirically for Baka Pygmies and Cameroon.

Model parameter	Baka Pygmies		Cameroon (countrywide)	
	Value	Reference	Value	Reference
Population size (N)	40,000	[35]	200,000	[41]
Life expectancy (Le) (years)	16.6	[52]	51.0	WHO^1^
Survivorship at age 5 (S_5_)	0.7	*estimated*	0.846	WHO
Survivorship at age 15 (S_15_)	0.4	[52]	0.823	WHO
Fraction born immune (NS)	0.15	[42]	0.15	[42]
*Transmission coefficients*		All derived from [41]		All derived from [41]
β_CC_	0.0000183		0.0000183	
β_CY_	0.00000367		0.00000367	
β_CA_	0.0000004		0.0000004	
β_YC_	0.00000367		0.00000367	
β_YY_	0.00000367		0.00000367	
β_YA_	0.0000004		0.0000004	
β_AC_	0.0000004		0.0000004	
β_AY_	0.0000004		0.0000004	
β_AA_	0.0000004		0.0000004	

1 WHO, 2012.

WHO Global Observatory Health Data Repository. World Health Organization, Geneva, Switzerland. http://apps.who.int/gho/data/. Last accessed on 11/02/2012.

N: For the Cameroon-wide simulation to validate the model, a population of 200,000 was adopted, following Rupnow *et al.* [41].

Le: For the Baka, the Migliano *et al.* estimate for the Aka was used [52].

S_5_: Fraction of individuals that reach five years of age. For the Baka, no estimate was available, so the base scenario assumes that half of mortality that will occur by age 15 takes place by age 5, in keeping with typically high infant mortality in developing-world contexts.

S_15_: Fraction of individuals that reach fifteen years of age. For the Baka, the Migliano *et al.* estimate for the Aka was used [52].

NS: Fraction born non-susceptible, i.e., the fraction of individuals that cannot become infected “for physiologic, physical, or immunologic reasons” [41]. Estimated from observed maximum prevalences in the developing world.

β_XY_: = Transmission coefficients, with X and Y in {C,Y, A}, where these stand for child, youth and adult age classes, respectively, yielding nine coefficients in all. This represents the probability of transmission from class X to class Y, and is derived from Rupnow *et al.* [41], as described in the text.

**Figure 3:**
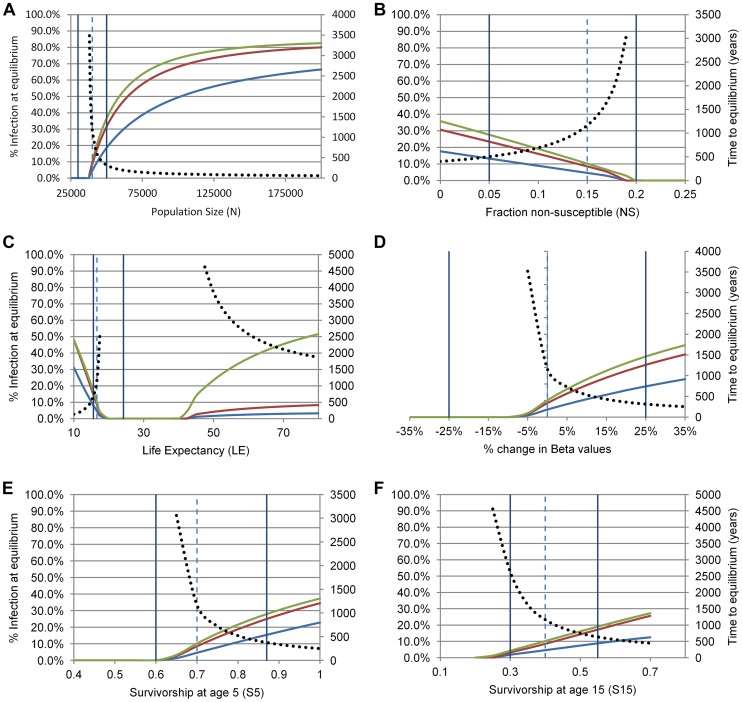
Sensitivity analysis of equilibrium values for *H. pylori* infection (primary y-axes) and time to equilibrium (secondary y-axes) in Baka Pygmies for six demographic and transmission parameters (panels A–F). For each panel, adults are in green, youths in red, children in blue. The dashed vertical line shows the value of the parameter adopted in the base scenario; the two solid vertical lines delimit plausible ranges of variation in this population, where these can be estimated from the literature. The dotted black line illustrates the time in years required for the prevalence in adults to approach to within 1% of the equilibrium value.

The model thus specified indicates a low equilibrium prevalence for the Baka of ∼10.2% under the assumptions of the base scenario. This is close to what we observed, and suggests that the time required to establish a stable equilibrium at this prevalence is considerably shorter than the estimated age of the most recent common ancestor of *H. pylori* haplotypes from Baka and non-Baka - under the base scenario the estimated time to equilibrium was 1,164 years. We tested the sensitivity of this model by varying the parameter estimates individually across a wide range of values, encompassing plausible ranges of demographic variation estimated from the literature or prior experience with the Baka (Figure 3). Equilibrium infection rates among adults remained low (<40%) over the ranges of plausible variation for individual parameters. Moreover, infection was nearly or entirely eliminated in the population for some values within these ranges for most parameters, suggesting that the Baka are near a limit for viable maintenance of population-level *H. pylori* infection. The sensitivity analysis indicated that the most crucial parameter was population size (Figure 3A). In fact, the observed infection rate of 20.8% is achieved at equilibrium in the model with a small population increase, to N = 43,000 from the 40,000 assumed in the base scenario. Mean life expectancy also appears to have a strong but non-linear effect on infection, with equilibrium prevalence falling to zero over intermediate values (Figure 3C). Time to equilibrium increased rapidly as equilibrium infection rates approached zero, decreasing the meaningfulness of this statistic at particularly low infection rates. However, within the range of uncertainty, time to equilibrium is reached in less than 1,000 years for those scenarios where the equilibrium infection rate is equal to or exceeds the value we observed in the Baka.

**Figure 4:**
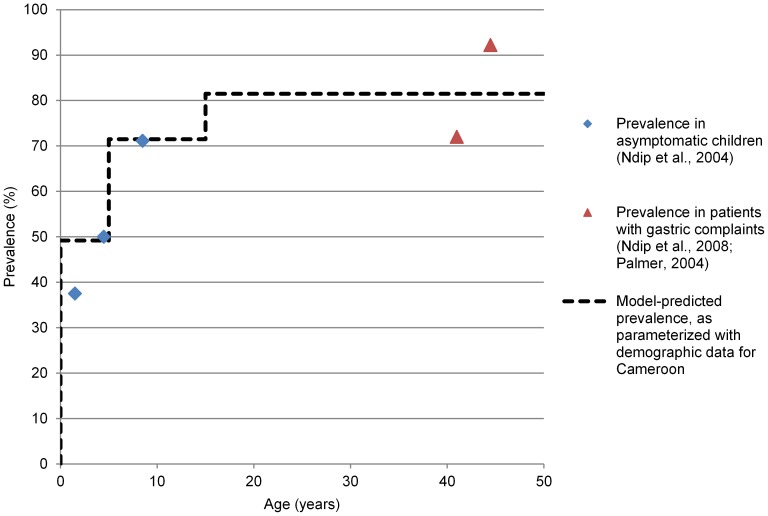
Model predicted prevalence under the base scenario versus observed *H. pylori* prevalences by age in Cameroon. The model-predicted prevalence rises in steps with the three age classes (i.e., child, youth, adult).

Increasing the population size to 200,000 and applying model parameters that are typical of Cameroon (Table 4) yielded infection frequencies that are consistent with non-Baka populations in Cameroon (Figure 4), and other parts of the developing world [42]. This supports the validity of the model and is consistent with the idea that human population sizes were fairly large during the 100,000 years that they have been infected by *H. pylori* [4].

As with all epidemiological models, our results should be interpreted with care. Following Rupnow *et al.* [41], we incorporated a frequency-dependent transmission parameter (rather than a density-dependent parameter). This is a common assumption for directly-transmitted diseases, but the estimated values from the original model might be inaccurate when applied to substantially different population sizes. Moreover, it is possible that another model structure might better capture the complex reality underlying *H. pylori* transmission, such as a model incorporating the possibility of environmental transmission. Although the utility of the original model in various contexts [41], [44], [45] suggests that it captures many of the most important aspects of transmission, further empirical work on the importance of environmental transmission in sub-Saharan Africa would allow us to refine our model accordingly. There is also uncertainty in parameter estimates. For example, the proportion of non-susceptible individuals can vary across populations as a function of innate immunity, lifestyle, contact patterns and/or factors such as atrophic gastritis which eliminates infection [42]. Our results rely on the accuracy of demographic and transmission estimates for the Baka, which in many cases are undefined or highly unusual for human populations. Further work verifying the accuracy of these estimates would be useful.

## Discussion

Based on the >100 kyr long association between *H. pylori* and man and our recent findings that the San hunter-gatherers of southern Africa were the original hosts of hpAfrica2 [4], we expected to identify unique *H. pylori* lineages that have long been associated with West African Baka Pygmies. Our results did not identify such lineages. Instead, *H. pylori* haplotypes from Baka belong to the hpAfrica1 or hpNEAfrica populations, which are also prevalent among other Africans (Figure 1A). Surprisingly, we also found that Baka were only rarely infected with *H. pylori*, significantly less than global frequencies in developing countries [6], [7], including their agriculturalist neighbors.

### Phylogeography

Under a *K* = 3 scenario, we identified a novel subpopulation within hpAfrica1, designated hspCAfrica, which because it comprises haplotypes from Cameroon and Angola, provides support for a Bantu agriculturalist migration along the western coast of Central Africa [30], [31] (see also Figure S1). Interestingly, we did not detect this population among 91 previously isolated hpAfrica1 strains from Namibia and South Africa, which were designated hspSAfrica and hspWAfrica (Figure 2B). This suggests that the western Bantu migration that brought hspCAfrica to Angola was not able to cross the Namib Desert and progress as far south as present-day South Africa. This agrees with the hypothesis that the Bantu who originally colonized South Africa were not part of this western migration, and instead brought hpAfrica1 to South Africa *via* the alternative eastern coastal route [31], [32]. We would therefore expect to find both hspSAfrica and hspWAfrica in high frequency among eastern African populations. Investigating the frequency of hpAfrica1 throughout south-central Africa could potentially shed even further light on the enigmatic Bantu migrations.

We also defined two subpopulations of hpNEAfrica, hspEastNEA and hspCentralNEA, which are distributed across the Sahel from Nigeria to Somalia. Sudan contains both subpopulations, suggesting that this subdivision may have originated there. Alternatively, the presence of both populations in Sudan may represent more recent, secondary migrations. The existence of hspCentralNEA in Cameroon, Nigeria and Algeria may correspond to the Holocene expansions that carried Nilo-Saharan languages from northeast Africa into the Sahara. We note however, that most of the Cameroonians from whom we isolated hpNEAfrica were Bantu speakers who presumably originated in West Africa. This situation resembles the frequent isolation of hpEurope and hpAfrica1 in South America, where they have largely displaced the ancestral hspAmerind bacteria [9], and the isolation of multiple populations of *H. pylori* from Australian aboriginals in addition to their ancestral hpSahul bacteria [10]. We also found that phylogenetic reconstructions of both hpAfrica1 (Figure S2) and hpNEAfrica (Figure S3) revealed greater detail about population structure than did Bayesian clustering. These showed a more gradual or gradient-like transition from one population/clade to another. Together, this evidence suggests that *H. pylori* is a sensitive indicator of human population admixture. Further genetic studies on populations in Cameroon may provide insights on local colonization processes, and the origins and diffusion of agriculturalist communities.

### Gene flow and contraction

The strong similarity of haplotypes from Baka Pygmies and non-Baka agriculturalists and the absence of significant differentiation between them justifies treating these *H. pylori* as a single metapopulation. This inference is plausible because Baka are in daily contact with their Bantu agriculturalist neighbors [46], and these populations exchange a low number of human migrants per generation [25]. Although we also inferred high gene flow between Baka and non-Baka populations [47], phylogenetic analyses suggest that Baka strains were derived from non-Baka ancestors. Under a hypothesis of acquisition of *H. pylori* by one group from another, we would expect that strains isolated from the donor population would occupy ancestral positions closer to the base of a subclade and that they would form closely related clusters, some strains of which would be passed on to the recipient population. This is precisely what we observe in both hpAfrica1 and hpNEAfrica phylogenies (Figures S2 and S3) - groupings among non-Baka strains, with one of the strains belonging to a Baka. We interpret these results, as well as the unusually low equilibrium prevalence of *H. pylori* among Baka, as reflecting the acquisition of *H. pylori* by the Baka *via* secondary contact with their non-Baka agriculturalist neighbors. This most likely occurred around 2,500 years ago with an upper bound of 3,973 years (Table 3). This age estimate for the Cameroon *H. pylori* metapopulation is in complete agreement with a hypothesis of recent secondary contact because Bantu expansions have been dated by archaeology to 3–5 kyr [34] and by human population genetics to ∼6 kyr [33]. Furthermore, the number of distinctly different and unrelated haplotypes found among the Baka is also suggestive of several independent agriculturalist to hunter-gatherer transmission events.

### Factors influencing *H. pylori* infection rate

According to our *H. pylori*-specific transmission model, the levels of infection observed in Baka can be achieved in a short timeframe after introduction into a naïve population, and are reproducible within the range of uncertainty in parameter estimates. The infection rate of 10.2% predicted by the model under the base scenario was slightly lower than the observed rate, possibly implying that the latter is influenced by continuously introduced infection from non-Baka agriculturalists. Regular and ongoing contact or cross-migration between Baka and agriculturalists would also imply a higher effective population size, which would increase the observed infection rate relative to that implied by the closed model. Conversely, we note that our samples were from Baka individuals with gastric complaints, which might overestimate the population prevalence of infection in the Baka (an effect that might be even more pronounced for non-Baka, who were sampled from among hospital patients).

Possibly the most striking finding from the simulations was that even small fluctuations in population size can result in the extinction of population-wide infection. This observation may explain the apparent absence of ancient bacterial lineages among Baka. While there are currently no estimates on the prehistoric census size of San or Pygmies, archaeological evidence suggests that San occupied a much wider territory than Pygmies [48]. From molecular demographic reconstructions, we know that prehistoric hunter-gatherer populations were larger [49], denser [50] and more constant in size [47] than in the present day, but they suffered declines during the Holocene at the advent of Neolithic agriculture. The Baka population size is now ∼40,000 [35], which is less than a fifth of the 208,000 extant San speakers [51]. Thus, the San, being less affected by the rise of agriculture, were able to retain their ancient *H. pylori* lineages, while any that existed among the Baka may have died out leaving an uninfected population which could acquire novel lineages from their non-Baka neighbors on secondary contact. The postulated ancient lineages might be found by more extensive sampling, and/or by sampling *H. pylori* from Eastern Pygmies. Alternatively, ancient haplotypes may never have existed and Baka were first infected with *H. pylori* in recent times.

A second interesting observation was that the short life expectancy among Pygmies [52] contributes to maintenance of endemic *H. pylori* infection. Transmission of *H. pylori* is particularly high during childhood [1], which in turn results in higher prevalence where life expectancy is low and children make up a larger proportion of the population. Our simulations indicate that the expected prevalence drops with increasing life expectancy - though it increases with age class, as individuals, once infected, remain infected. Intriguingly, prevalence rises once more when life expectancy exceeds 40 years, despite lower rates of transmission in adults. This is probably because longer-lived adults can potentially transmit infections for much longer periods. However, these insights were obtained by varying single parameters while holding other variables constant at base-scenario levels; simultaneous fluctuation in several parameter values would result in a wider range of estimated endemic prevalence values.

Apart from violations of model assumptions, other explanations could potentially also account for the low prevalence of *H. pylori* among the Baka, including their hunter-gatherer diet based on forest products and the medicinal use of rain-forest plants with antibiotic properties [53]–[55]. A low prevalence of *H. pylori* in Java, Malaysia and Tanzania has also been attributed to local diet [56] but those epidemiological data have not been considered in light of a demographic transmission model as presented here [57]. Finally, genetic or innate immunity that is specific to the Baka may also have played a role.

## Materials and Methods

### Study area, sample selection, and ethics statement

We undertook field collection of gastric biopsies at two settlements in the rainforests of the East Province of Cameroon. Yokadouma and Abong Mbang are towns of more than 20,000 inhabitants that lie about 250 km apart. The Bantu populations are engaged mainly in primary agriculture (hereafter referred to as agriculturalists), while the Baka are hunter-gatherer communities that live in the forests surrounding both settlements. Study participants were selected from among individuals complaining of gastric pain. Esophagogastroduodenoscopy was performed by a certified gastroenterologist (A.M.) with written informed consent at Yokadouma and Abong Mbang Hospitals under ethics certificate 0002/ERCC/CBNO2 from the Cameroon Bioethics Initiative (CAMBIN), administrative authorization 631.19-09 from the Cameroon Ministry of Public Health (Secretariat General, Division of Health Operations Research), and with permission of the ethics committee of the Charité Hospital in Berlin, Germany (ethics certificate EA1/071/07). Among agriculturalists, samples were taken from patients at their respective district hospitals, whereas Baka - who were unlikely to attend the hospital - were recruited directly from their communes by an NGO worker familiar with the area. Baka communes were provided minor food incentives (e.g., rice) for participation, which did not depend on the number of participants from each village. Non-participation or refusal was rare among approached subjects, both at the hospital and in the field.

### *H. pylori* samples and culture

Gastric biopsies were taken from 178 Cameroonians belonging to 25 different ethnic groups (Table 1). All individuals were endoscopically evaluated for gastroduodenal pathology, histological evaluation was not performed. Biopsies were obtained from both the *antrum* and *corpus* of the stomachs of 176 people, while only single biopsies from the *corpus* were obtained from two individuals. All biopsies were immersed in PBS (phosphate buffered saline) and immediately frozen in liquid nitrogen, kept at −80°C and couriered to the Hannover Medical School. *H. pylori* was cultivated by inoculation of the biopsies on blood agar plates (Columbia-Agar-Basis II, Oxoid, Wesel) supplemented with 10% horse blood (Oxoid), and the antibiotics vancomycin (10 mg/l), polymyxin B (2500 U/l), amphotericin B (4 mg/l) and trimethoprim (5 mg/l). Plates were incubated under microaerobic conditions (5% O_2_, 10% CO_2_, 85% N_2_) for 3 to 5 days at 37°C. In case of lack of bacterial growth, the plates were incubated for longer periods of time. Since concomitant infection with several different *H. pylori* strains has been previously observed [2], strains were purified by single colony isolation from one colony from each of the *antrum* and *corpus* biopsies, resulting in a total of 191 isolates. To ensure optimal efficiency of cultures, the entire biopsies were used for culture, and *antrum* and *corpus* biopsies were cultured by independent investigators on separate days, with overall excellent agreement between results.

### Molecular genetics

Genomic DNA of bacterial strains was isolated using the QIAamp DNA Mini Kit (Qiagen). Seven housekeeping gene fragments (*atpA*, *efp*, *mutY*, *ppa*, *trpC*, *ureI*, *yphC*) were amplified by PCR. Primer sequences and primer combinations can be found at http://pubmlst.org/helicobacter. PCR was performed under the following conditions: initial denaturation of 5 min at 94°C, 30 cycles of 1 min at 94°C, 1 min of annealing, 1 min at 72°C, and a final extension of 7 min at 72°C. The annealing temperature was either 57°C (*atpA*, *trpC*), 55°C (*yphC*), 53°C (*efp*, *mutY*), or 51°C (*ppa*, *ureI*). PCR products were purified using the QIAquick 96 PCR Purification Kit (Qiagen). PCR amplicons were sequenced bidirectionally using the BigDye Terminator v1.1 Cycle Sequencing Kit and the 3130xl Genetic Analyzer (Applied Biosystems). Sequence data were analyzed using BioNumerics v6.01 (Applied Maths NV, Sint-Martens-Latem, Belgium).

### Diversity and structure

Data graphing and statistical analyses were performed with Origin 8.0 (OriginLab, USA) and SigmaStat 3.5 (SYSTAT, USA). Population genetic diversity indices were calculated using DnaSP v5 [58].

A test of population subdivision was applied to evaluate whether Baka and non-Baka *H. pylori* populations were panmictic [59]. This statistic is particularly effective in detecting population structure in the presence of high recombination, as is the case for *H. pylori*. The test generated a random distribution for the Ks statistic (sum of the mean number of pairwise differences for each population weighted by population) using a Monte Carlo approach that randomized genotypes between the two populations using 10,000 simulations. The null hypothesis of panmixia is not rejected if p(Ks)>0.05.

We performed Bayesian population assignments on the 113 Cameroonian *H. pylori* haplotypes along with another 792 previously published global reference haplotypes [8]–[10]. The “no admixture model” and “linkage model” of the program STRUCTURE 2.0 [39] were used to assign the Cameroonian isolates to one of the known modern *H. pylori* populations [8]–[10] and to explore their level of reciprocal admixture, respectively. In order to determine the number of bacterial populations (*K*) within the dataset ten independent STRUCTURE runs of 50,000 iterations following a burn-in period of 25,000 iterations for each *K* value were analyzed and compared for consistency. To reveal subpopulation structure subsequent analyses were performed separately for hpAfrica1 and hpNEAfrica isolates with ten independent STRUCTURE runs for each *K* value. The Cameroonian isolates assigned to hpAfrica1 (n = 44) were further analyzed together with 93 hspSAfrica and 73 hspWAfrica reference isolates. The Cameroonian isolates assigned to hpNEAfrica (n = 67) were further analyzed together with 126 hpNEAfrica reference isolates. The latter include 65 previously published haplotypes [8], [9] as well as 61 unpublished haplotypes obtained from individuals in Sudan in 2004 and 2005. Culture and sequence analysis of the Sudanese isolates were performed as described above for the samples from Cameroon. The STRUCTURE runs yielding the highest model log-likelihood were graphically displayed using the program DISTRUCT [60].

The software ClonalFrame v1.1 [61] was used to determine ancestral relationships among strains within the hpAfrica1 and hpNEAfrica populations. ClonalFrame reconstructs phylogenetic genealogies while accounting for horizontal gene transfer and its value in consistently reconstructing *H. pylori* phylogenies has been demonstrated previously [4], [10]. The program was run with 100,000 MCMC iterations with a thinning interval of 100 after an initial burn-in phase of 50,000 iterations. Ten independent ClonalFrame runs were performed. Using Treefinder [62] an 80% consensus tree of all independent ClonalFrame runs was computed. Three hpAfrica2 haplotypes were used as an outgroup to root both hpAfrica1 and hpNEAfrica trees.

### Gene flow and coalescence

We used the software Jaatha 2.0 [40] to estimate the time to population split and the level of gene flow between Baka and non-Baka *H. pylori* populations. This coalescence-based software uses the observed joint allele frequency spectrum as a summary statistic to estimate the composite likelihood of posterior parameters in user-defined demographic models. This flexibility allows the user to control for between-population differences in demographic history by explicitly simulating either constant or expanding populations. Furthermore, Jaatha is also able to model recombination within the coalescent framework, controlling for horizontal gene transfer, and is particularly robust in estimating the time to population split in cases of recent population divergence [40], as may be likely among the studied populations. We ran two models: the first assuming that the non-Baka bacterial population underwent exponential growth and Baka remained constant in size; and the second with both populations evolving under exponential growth. These two settings approximate well the possible demographic histories of the two bacterial populations based on previous knowledge of host demographic history. As a first step, we used wide prior values (see Table S2) to explore the parameter probability space for the best starting point for a refined estimate of the posterior parameters. The probability space was divided into a number of equal blocks in which coalescent simulations using the software ms [63] were performed. Since we simulated sets of 6 and 7 parameters for Baka constant and expansion models, probability space was divided into 3^6^ and 3^7^ blocks, respectively, for each of which 200 simulations were performed using the range of prior parameter values. The joint allele frequency spectra were divided into 23 independent Poisson-distributed values and fitted using a generalized linear model. A score proportional to the likelihood was calculated for each parameter set and those with the two highest scores were used as starting points in the final search. Then, a search of 10,000 simulations was repeated 200 times for the two best starting points with five independent seeds. The 10 best parameter combinations for each of these 10 simulations were then subjected to a further simulation step of 100,000 iterations from which the composite likelihood of each parameter combination was calculated. The distribution of posterior parameters was used to estimate confidence intervals. The times to population split in years were calculated using a mutation rate (μ = 2.09×10^−6^), which is the average of the three estimates presented in Morelli *et al.* [64].

### Transmission model

To estimate the length of time needed to reach equilibrium *H. pylori* infection among Baka Pygmies, we applied a simple age-structured transmission model, modified from Rupnow *et al.* [41]. The model assumes a standard Susceptible-Infected (SI) structure dividing the model population into three age groups, namely, children (0–5 years old), youths (5–15 yo) and adults (15+ yo). For a description of model parameters see Table 4.

We constructed a base scenario using the best available estimates for the demographic and transmission parameters: life expectancy (Le), population size (N), survivorship at age 5 (S_5_), survivorship at age 15 (S_15_), and fraction of individuals born into the non-susceptible class (NS). Since demographic variables for Western Pygmies are largely uncharacterized [65], we used parameters from a Pygmy population adjacent to the Baka (the Aka) [52] where available; during sensitivity analysis we included the entire range of plausible parameter variation, estimated from the literature where possible. In contrast to the model by Rupnow *et al.* [41], we allowed for inter-age class transmission. To obtain approximate values for the resulting nine transmission constants (β), we divided those obtained for the prior model - i.e., for intra-age class transmission - by three, and assumed that transmission between age groups was governed by the characteristics of the less-transmissive group. To test the validity of the β-values thus specified, we applied them to a simulation using a population of 200,000 (following Rupnow *et al.* [41]) and demographic parameters for Cameroon. Because the results fit well with prior observations of age-specific prevalence (see Figure 4), we retained these values for the base scenario.

To test the sensitivity of results to changes in input parameters, we varied each parameter incrementally, while holding all others at base scenario values. When varying transmission constants, we assumed that the relative strength of transmission among age groups remains constant. Simulations were run until the system reached equilibrium with a time step (dt) of 0.25 years, using the Runge-Kutta 4 integration method in iThink 7.0.3 (High Performance Systems, Inc., Hanover, NH).

### Data deposition

This publication made use of the *Helicobacter pylori* Multi Locus Sequence Typing website (http://pubmlst.org/helicobacter/) developed by Keith Jolley and sited at the University of Oxford [66]. The development of this site has been funded by the Wellcome Trust and European Union. Each new isolate from Cameroon and Sudan was assigned an ID number (1663–1836).

## Supporting Information

Figure S1 Major human population events in recent African prehistory. This map summarises the potential major human demographic events as inferred from [4], [20], [25], [27], [28], [33], [47], [67]. Ancestral hunter-gatherer populations split into extant populations, beginning with Northern and Southern San (32–47 kya) and Western and Eastern Pygmies ∼20 kya. The Nilo-Saharan speakers originated in northeast Africa from where they migrated both north-westward and south-eastward. Their north-westward migration was favored by a climatic change from dry to more humid conditions during the humid Holocene period (6–9 kya). Bantu-speaking agriculturalist populations expanded from their homeland in what is present-day Nigeria/Cameroon, beginning ∼6 kya, in two independent waves along the western and eastern flanks of Africa into southern Africa. They thus spread their languages and agriculture through most of sub-Saharan Africa within the last 5 kyr.
(TIF)

Figure S2 Phylogenetic relationships among hpAfrica1 isolates. Phylogenetic relationships among hpAfrica1 isolates as determined by ClonalFrame. The haplotypes are colored according to their geographical source and symbols (triangles, circle) specify the respective subpopulation determined by STRUCTURE. Cameroonian haplotypes from non-Baka (brown color) or Baka (pink color) are marked. The hpAfrica2 strains used as outgroup to root the tree are separately indicated. The number of isolates is shown in brackets.
(PDF)

Figure S3 Phylogenetic relationships among hpNEAfrica isolates. Phylogenetic relationships among hpNEAfrica isolates as determined by ClonalFrame. The color-code corresponds to the geographical source. Cameroonian haplotypes isolated from non-Baka (brown color) or Baka (pink color) are indicated. One haplotype was isolated from both non-Baka and Baka individuals (dark-green color). The hpAfrica2 strains used as outgroup to root the tree are separately indicated. The number of isolates is shown in brackets.
(PDF)

Table S1 Frequencies of endoscopy findings in Baka Pygmy and non-Baka agriculturalist study participants.
(DOC)

Table S2 Prior parameters used in coalescent simulations with Jaatha 2.0 and their values.
(DOC)
