# Supplementary figures and images for: Recent Acquisition of Helicobacter pylori by Baka Pygmies
Source: PLoS Genet. 2013 Sep 19;9(9):e1003775. doi: 10.1371/journal.pgen.1003775 (PMC3777998; doi:10.1371/journal.pgen.1003775)

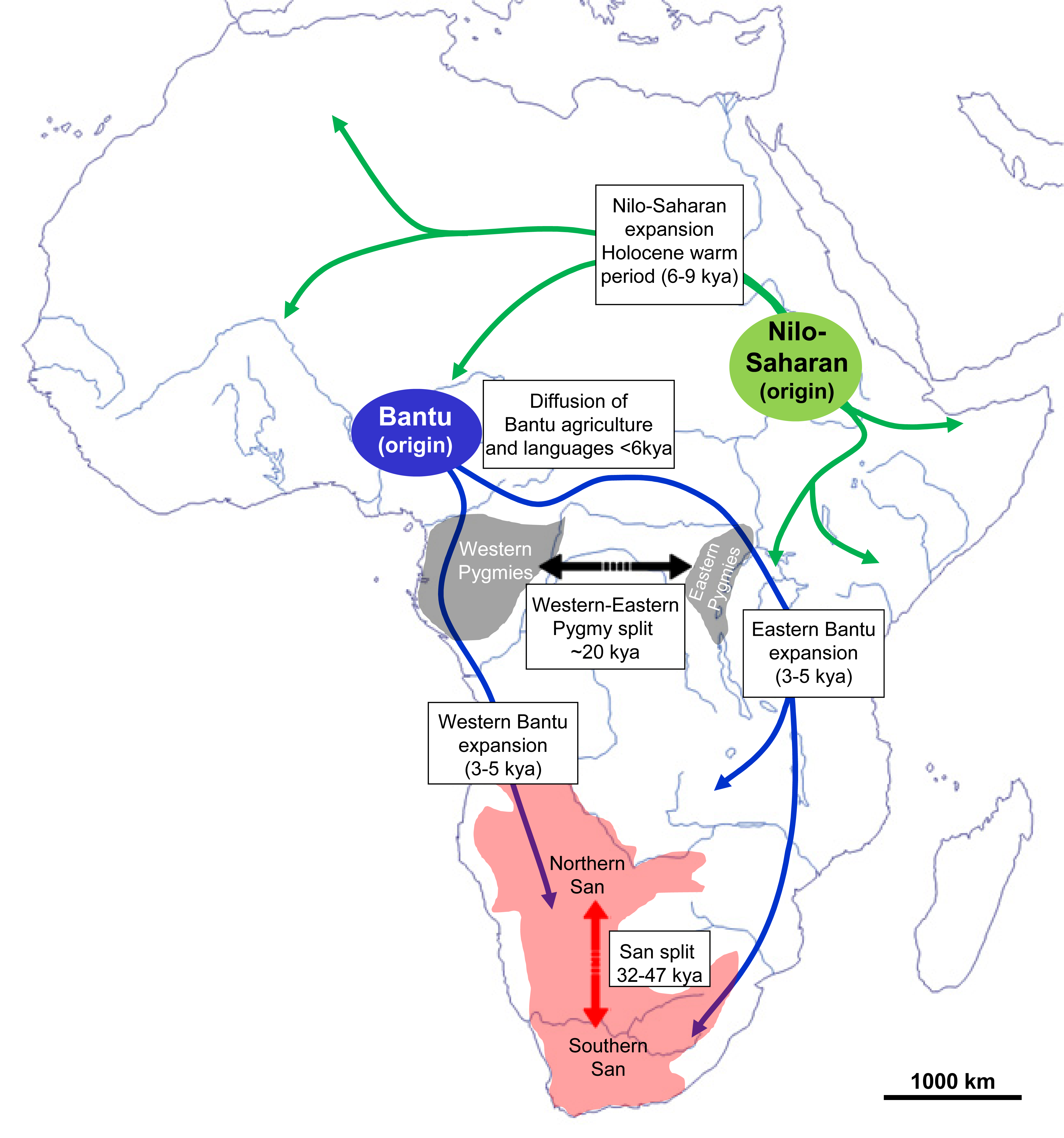

Supplement: Figure S1 — Major human population events in recent African prehistory. This map summarises the potential major human demographic events as inferred from [4], [20], [25], [27], [28], [33], [47], [67]. Ancestral hunter-gatherer populations split into extant populations, beginning with Northern and Southern San (32–47 kya) and Western and Eastern Pygmies ∼20 kya. The Nilo-Saharan speakers originated in northeast Africa from where they migrated both north-westward and south-eastward. Their north-westward migration was favored by a climatic change from dry to more humid conditions during the humid Holocene period (6–9 kya). Bantu-speaking agriculturalist populations expanded from their homeland in what is present-day Nigeria/Cameroon, beginning ∼6 kya, in two independent waves along the western and eastern flanks of Africa into southern Africa. They thus spread their languages and agriculture through most of sub-Saharan Africa within the last 5 kyr. (TIF) [file pgen.1003775.s001.tif]

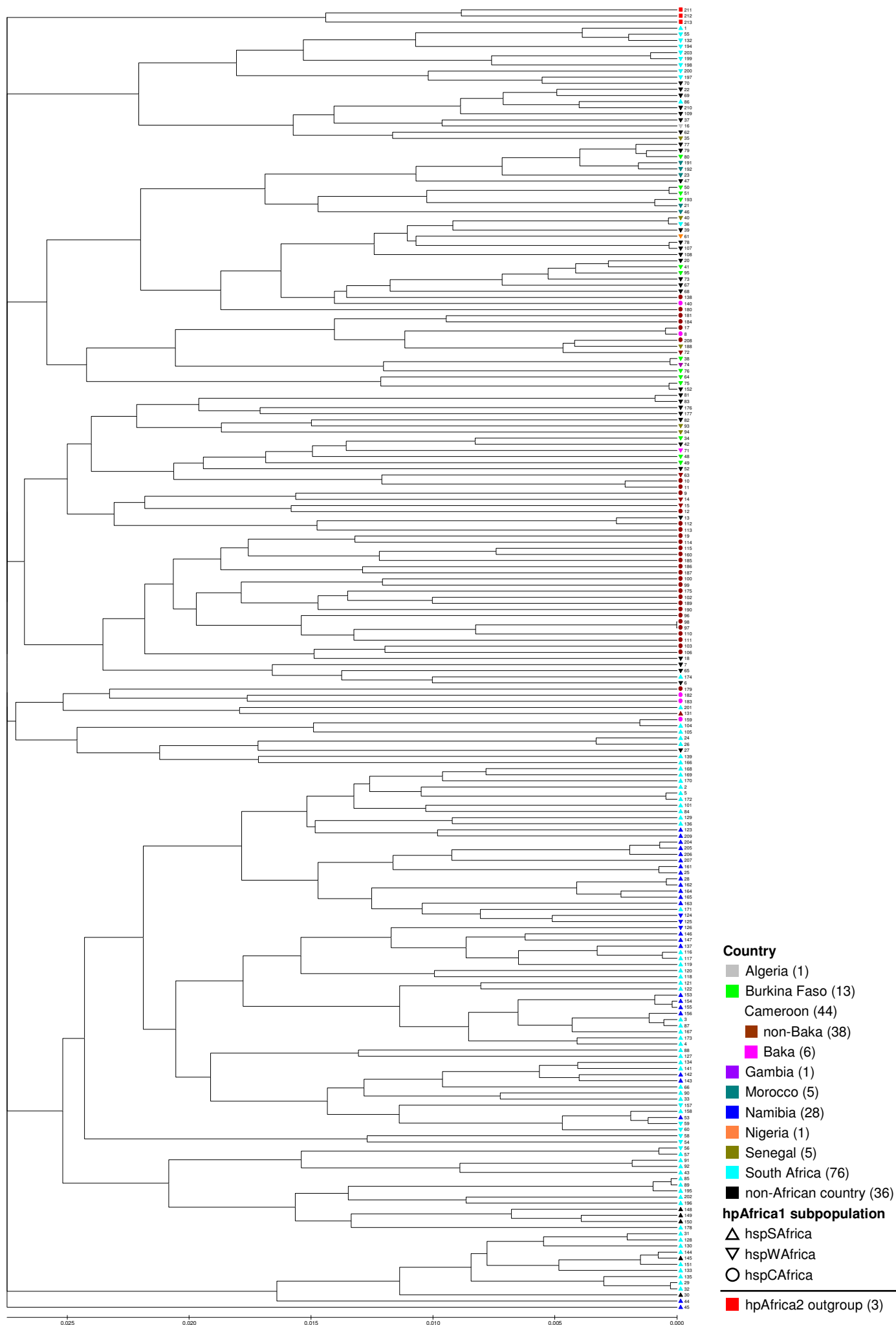

Supplement: Figure S2 — Phylogenetic relationships among hpAfrica1 isolates. Phylogenetic relationships among hpAfrica1 isolates as determined by ClonalFrame. The haplotypes are colored according to their geographical source and symbols (triangles, circle) specify the respective subpopulation determined by STRUCTURE. Cameroonian haplotypes from non-Baka (brown color) or Baka (pink color) are marked. The hpAfrica2 strains used as outgroup to root the tree are separately indicated. The number of isolates is shown in brackets. (PDF) [file pgen.1003775.s002.pdf]

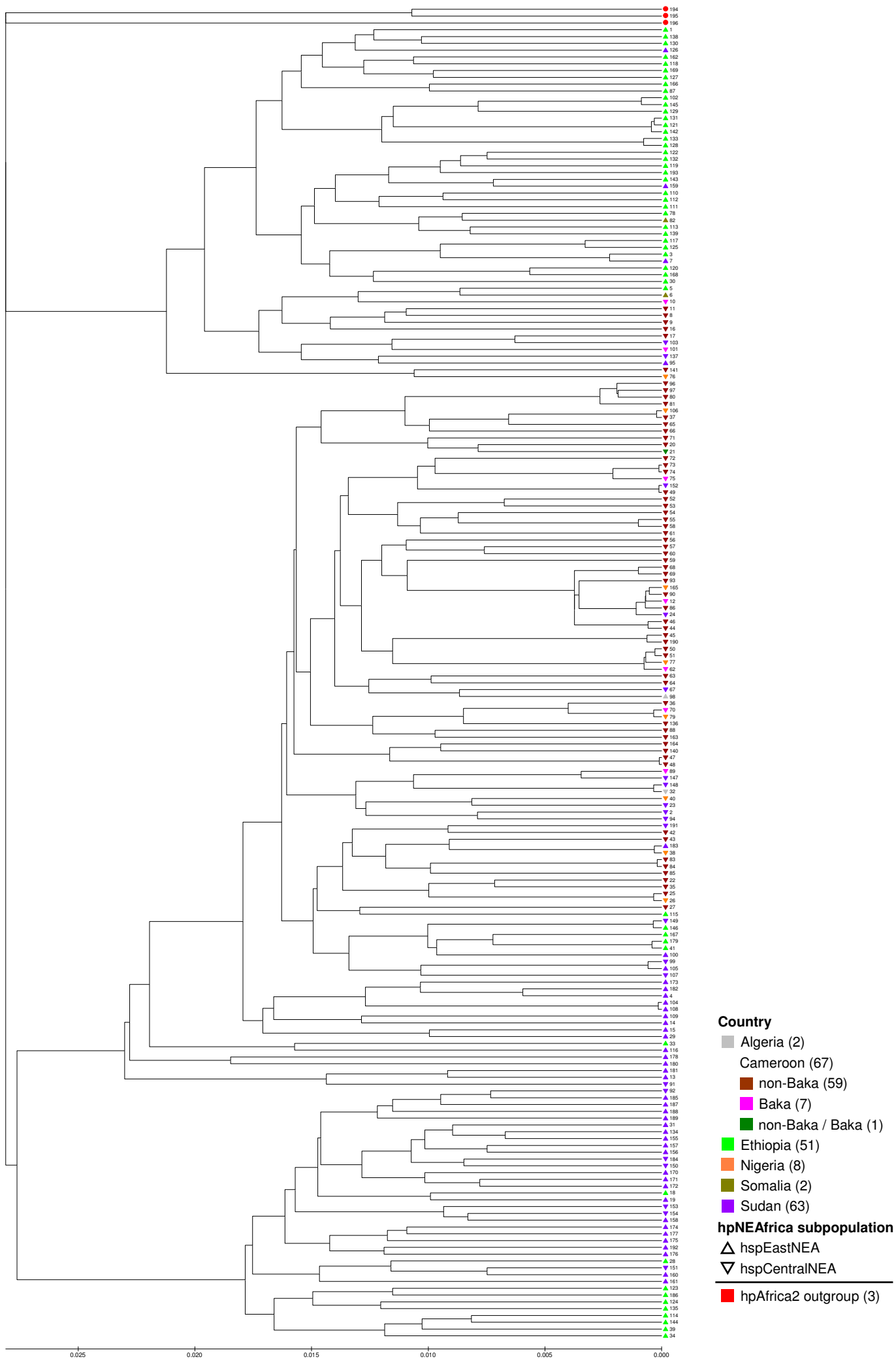

Supplement: Figure S3 — Phylogenetic relationships among hpNEAfrica isolates. Phylogenetic relationships among hpNEAfrica isolates as determined by ClonalFrame. The color-code corresponds to the geographical source. Cameroonian haplotypes isolated from non-Baka (brown color) or Baka (pink color) are indicated. One haplotype was isolated from both non-Baka and Baka individuals (dark-green color). The hpAfrica2 strains used as outgroup to root the tree are separately indicated. The number of isolates is shown in brackets. (PDF) [file pgen.1003775.s003.pdf]
