# Supplementary material for: Recent Acquisition of Helicobacter pylori by Baka Pygmies
Source: PLoS Genet. 2013 Sep 19;9(9):e1003775. doi: 10.1371/journal.pgen.1003775 (PMC3777998; doi:10.1371/journal.pgen.1003775)
Supplement: Table S1 — Frequencies of endoscopy findings in Baka Pygmy and non-Baka agriculturalist study participants. (DOC) [file pgen.1003775.s004.doc]

**Table S1.** Frequencies of endoscopy findings in Baka Pygmy and non-Baka agriculturalist study participants.

| **Endoscopic finding** | **No. of Baka** | | | **No. of non-Baka** | | |
| --- | --- | --- | --- | --- | --- | --- |
|  | HP pos | HP neg | total | HP pos | HP neg | total |
| No pathology | 5 | 15 | 20 | 13 | 3 | 16 |
| Chronic gastritis | 8 | 20 | 28 | 18 | 2 | 20 |
| Duodenal ulcer | 1 | 1 | 2 | 7 | 0 | 7 |
| Erosive gastritis | 0 | 3 | 3 | 15 | 2 | 17 |
| Multiple gastric polyps | 0 | 1 | 1 | 0 | 0 | 0 |
| Post-ulcer scar in duodenum | 0 | 0 | 0 | 3 | 0 | 3 |
| Reflux | 1 | 5 | 6 | 6 | 4 | 10 |
| Superficial gastritis | 1 | 16 | 17 | 18 | 9 | 27 |
| Superficial gastritis + Reflux | 0 | 0 | 0 | 1 | 0 | 1 |
