# Supplementary material for: Recent Acquisition of Helicobacter pylori by Baka Pygmies
Source: PLoS Genet. 2013 Sep 19;9(9):e1003775. doi: 10.1371/journal.pgen.1003775 (PMC3777998; doi:10.1371/journal.pgen.1003775)
Supplement: Table S2 — Prior parameters used in coalescent simulations with Jaatha 2.0 and their values. (DOC) [file pgen.1003775.s005.doc]

**Table S2**. Prior parameters used in coalescent simulations with Jaatha 2.0 and their values.

| Parameter | Minimum value | Maximum value |
| --- | --- | --- |
| Theta *θ* | 1 | 100 |
| Divergence *τ* | 0.0001 | 2 |
| Migration *m* | 0.001 | 2 |
| Growth *α* | 0.0001 | 0.01 |
| Recombination *rho* | 0.0001 | 2 |

*θ =* 4N_1_*μ* where N_1_ is the effective population size of the reference population (non-Baka). Given the estimate of the mutation rate for *Helicobacter pylori* and bacteria in general (Morelli *et al.*, 2010), the prior interval spans ~2e5 to ~23e6.

*τ*, the time to population split in coalescent units. Time in years was estimated as *τ∙*4N_1_. Thus the minimum time allowed is ~120 years

*m =* migration rate is the fraction of incoming immigrants per generation into each population. As *θ* is explicitly estimated only in the reference population (Baka), we were not able to deduce the actual number of effective migrants (*θ*m) between populations.

α = population growth rate. This was fixed at 0 for the Baka population in model 1, where its population size was constant.

*rho =* the recombination rate.

Priors for both migration and recombination were initially set to 0.1-20, but were reduced after several trial runs yielded low posterior estimates. Migration and growth rate priors were the same for both populations.
